# Multimodal learning system integrating electronic medical records and hysteroscopic images for reproductive outcome prediction and risk stratification of endometrial injury: a multicenter diagnostic study

**DOI:** 10.1097/JS9.0000000000001241

**Published:** 2024-03-11

**Authors:** Bohan Li, Hui Chen, Xiaona Lin, Hua Duan

**Affiliations:** a Department of Minimally Invasive Gynecologic Center, Beijing Obstetrics and Gynecology Hospital, Capital Medical University, Beijing Maternal and Child Health Care Hospital; b School of Biomedical Engineering; c Beijing Advanced Innovation Center for Big Data-based Precision Medicine, Capital Medical University, Beijing; d Assisted Reproduction Unit, Department of Obstetrics and Gynecology, Sir Run Run Shaw Hospital, School of Medicine, Zhejiang University, Key Laboratory of Reproductive Dysfunction Management of Zhejiang Province, Hangzhou, People’s Republic of China

**Keywords:** conception, electronic medical records, hysteroscopy, intrauterine adhesions, multimodal learning

## Abstract

**Objective:**

To develop a multimodal learning application system that integrates electronic medical records (EMR) and hysteroscopic images for reproductive outcome prediction and risk stratification of patients with intrauterine adhesions (IUAs) resulting from endometrial injuries.

**Materials and methods:**

EMR and 5014 revisited hysteroscopic images of 753 post hysteroscopic adhesiolysis patients from the multicenter IUA database we established were randomly allocated to training, validation, and test datasets. The respective datasets were used for model development, tuning, and testing of the multimodal learning application. MobilenetV3 was employed for image feature extraction, and XGBoost for EMR and image feature ensemble learning. The performance of the application was compared against the single-modal approaches (EMR or hysteroscopic images), DeepSurv and ElasticNet models, along with the clinical scoring systems. The primary outcome was the 1-year conception prediction accuracy, and the secondary outcome was the assisted reproductive technology (ART) benefit ratio after risk stratification.

**Results:**

The multimodal learning system exhibited superior performance in predicting conception within 1-year, achieving areas under the curves of 0.967 (95% CI: 0.950–0.985), 0.936 (95% CI: 0.883–0.989), and 0.965 (95% CI: 0.935–0.994) in the training, validation, and test datasets, respectively, surpassing single-modal approaches, other models and clinical scoring systems (all *P*<0.05). The application of the model operated seamlessly on the hysteroscopic platform, with an average analysis time of 3.7±0.8 s per patient. By employing the application’s conception probability-based risk stratification, mid-high-risk patients demonstrated a significant ART benefit (odds ratio=6, 95% CI: 1.27−27.8, *P*=0.02), while low-risk patients exhibited good natural conception potential, with no significant increase in conception rates from ART treatment (*P*=1).

**Conclusions:**

The multimodal learning system using hysteroscopic images and EMR demonstrates promise in accurately predicting the natural conception of patients with IUAs and providing effective postoperative stratification, potentially contributing to ART triage after IUA procedures.

## Graphical abstract

**Figure:**
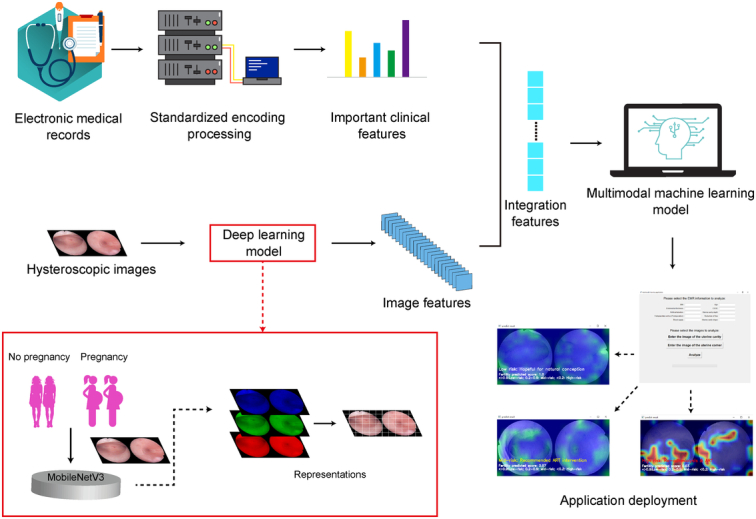

## Introduction

Highlights

- A multimodal learning application system was developed and validated using a combined electronic medical records and hysteroscopic images. It was applied to predict reproductive outcomes in a multicenter cohort of 753 Intrauterine adhesions.
- The multimodal learning model, constructed using hysteroscopic images and electronic medical records, demonstrated significant clinical potential in accurately predicting natural conception ability.
- Utilizing the system’s predicted probabilities of reproductive outcomes for risk stratification provides a potential reference value for postoperative assisted reproductive technology triage.

Intrauterine adhesions (IUAs), also known as Asherman’s syndrome, result from injury to the endometrial basal layer and subsequent scar formation^1^. IUAs often occur secondary to induced abortions or intrauterine procedures and are more common in developing countries, with incidence rates of up to 14% in patients with infertility or recurrent pregnancy loss^2,3^. Hysteroscopic adhesiolysis is currently the preferred treatment for IUAs owing to its minimally invasive nature and direct visualization^4–6^. However, managing moderate-to-severe IUAs remains challenging, and severe cases are associated with poor prognosis^2^. Considering the impact of IUAs on natural fertility, assisted reproductive technology (ART) is commonly used to facilitate pregnancy outcomes^7^. However, the costs are considerably high, especially in developing countries^8,9^. Few studies have explored effective postoperative ART triage strategies for IUAs, to identify populations facing difficulties in achieving natural conception, establish risk-stratified post-IUA management, and balance patient costs, efficacy, and time^10,11^.

Previous indicators for evaluating IUAs include clinical score systems rated by organizations such as the American Fertility Society (AFS), European Society of Gynecological Endoscopy (ESGE), and Chinese Society of Gynecological Endoscopy (CSGE)^12–14^. However, their subjective nature often yields varying efficacies across different studies^2^. Multimodal learning is a recent prominent trend in medical artificial intelligence (AI) development^15,16^. It combines various data sources for disease diagnosis and prognosis, including imaging and electronic medical records (EMR). Multimodal learning overcomes the potential data biases associated with single-modal approaches by analyzing details from different data modalities and has significant application potential^17^. This study hypothesized that a multimodal learning application system based on hysteroscopic images and EMR could effectively predict the likelihood of natural conception within a year and stratify the subfertility risk of patients with IUAs, thus enabling postoperative ART triage and risk stratification.

## Methods

### Participant recruitment

Data for this study were sourced from the Chinese Multicenter Intrauterine Adhesions Cohort Database^10^. A total of 1016 patients from two research centers in Beijing (*n*=712) and Zhejiang (*n*=304) with hysteroscopy-confirmed adhesions were prospectively enrolled between December 2018 and January 2020. The inclusion criteria were as follows: 1) patients with hysteroscopy-confirmed adhesions and a history of infertility or recurrent pregnancy loss (≥2 early pregnancy losses); 2) patients aged 20–45 years with a desire for conception; 3) patients devoid of gynecological endocrine disorders (e.g. polycystic ovary syndrome) and with antiMüllerian hormone levels >1 ng/ml; 4) male partners exhibiting no sperm abnormalities; and 5) patients who provided informed consent. A total of 753 individuals met the criteria. All participants provided written informed consent for the collection and use of their clinical samples and medical data. The follow-up period was 1-year, and the endpoint was defined as achieving an ongoing pregnancy, confirmed ultrasonographically after 12 weeks of gestation. The primary outcome was the prognosis of conception within 1-year. This study was reported according to the extension guidelines of STARD (Supplemental Digital Content 1, http://links.lww.com/JS9/C24) (Appendix PP 1–4)^18^ (Supplemental Digital Content 2, http://links.lww.com/JS9/C25).

### Hysteroscopic adhesiolysis and data collection

All 753 patients underwent hysteroscopic adhesiolysis. The procedure started with preoperative cervical softening using a derivative of prostaglandin F2α (PGF2α). The uterine cavity and adhesions were assessed using surgical hysteroscopy and saline perfusion. Precise tissue separation and scar removal preserved the endometrium with the aim of restoring the uterine anatomy. Postoperatively, physical barriers prevented adhesion recurrence. A 3-month hormone therapy regimen comprising estradiol valerate and dydrogesterone was administered. Patients were advised to attempt natural conception for one year, and ART was recommended if they were unsuccessful. A three-cycle regimen involving exogenous estrogen and progestogen was administered. After treatment, hysteroscopic re-examination was performed to capture intrauterine images that were subsequently incorporated into the analytical framework. Comprehensive clinical information encompassing preoperative (22 items) and postoperative (6 items) variables was systematically compiled for all patients (Appendix PP 5–7, Supplemental Digital Content 2, http://links.lww.com/JS9/C25). Follow-up data were obtained through phone calls or clinical records.

### Ethical approval

Permission to conduct the study was granted, and the study was supervised by the Research Ethics Committee.

### Establishing, evaluating, and applying the multimodal learning model

#### Patients assignments

**Figure 1:**
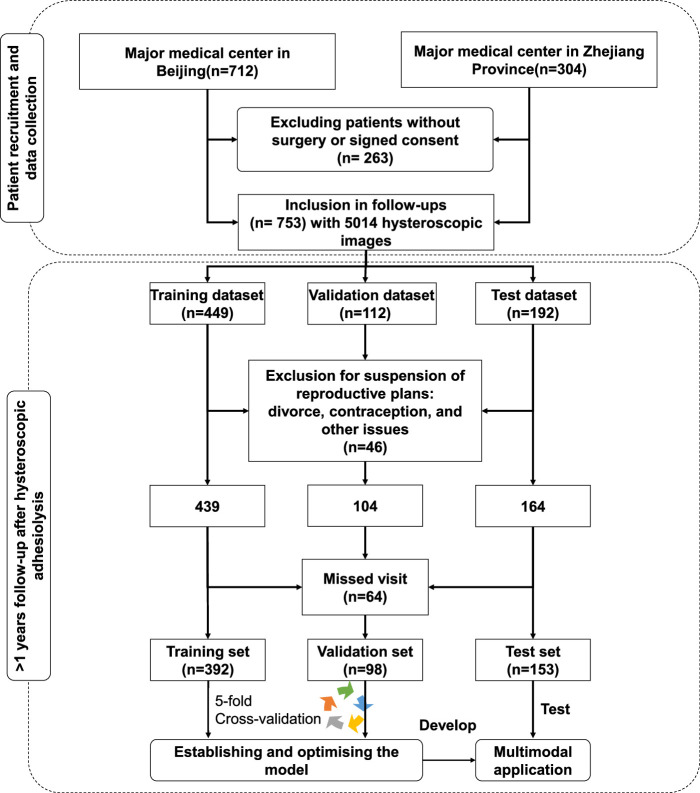
Flow diagram of the cohort study.

The research cohort was randomly split into three datasets: 60% for training, 15% for validation, and 25% for testing. The training dataset was used to build the initial predictive model, the validation dataset was used to optimize the hyperparameters, and the test dataset was used to externally validate the generalization performance of the application developed using the multimodal model. The test dataset was not involved in the model development or tuning stage (Fig. 1).

#### EMR feature extraction

The most predictive clinical features were initially selected by conducting Cox univariate regression analysis on the training dataset and identifying features with *P*-values <0.1. Subsequently, the predictive capabilities of the selected EMR features were systematically evaluated using the XGBoost algorithm, a gradient boosting method that can handle both numerical and categorical data. The relative importance of each feature was calculated, and the minimum number of features required for acceptable accuracy was identified. Model interpretability and simplicity were enhanced using the features with the highest importance and by assessing the concordance index (C-index)^19^.

#### Hysteroscopic image feature extraction

The process of hysteroscopic image acquisition is detailed in Appendix PP 8–9 (Supplemental Digital Content 2, http://links.lww.com/JS9/C25). By leveraging the efficiency of the MobilenetV3 framework, image features were extracted to capture the relevant information. Each patient’s dataset was represented by two images, each resized to 336×336 pixels using the Keras preprocessing image package. Convolution kernel weights were harnessed to extract hierarchical features from diverse layers. A dropout rate of 0.2 was applied to mitigate overfitting during feature extraction. The dense-layer architecture optimized through hyperparameter tuning facilitated the extraction of representative features.

#### Subfertility risk visualization

Gradient-weighted Class Activation Mapping (Grad-CAM) is a robust technique for constructing visual explications within the context of predictive modeling. This method leverages gradients originating from an arbitrary target concept that are subsequently propagated through convolutional layers to engender a rudimentary localization map. This map accentuates salient regions within an image that are significant for the prediction of a given concept fertility outcomes prediction^20^.

In this study, used Grad-CAM to elucidate the interpretability of the outcomes of our AI model. We aimed to elucidate the discerning attributes and discriminating features that the model relies upon when delineating between favorable and unfavorable pregnancy instances, thereby enabling the identification and exposition of regions of interest (ROIs), as deduced by automated analysis facilitated by the AI application.

#### Establishment and tuning of multimodal learning

The crux of our approach lies in establishing and optimizing a multimodal learning model. By synergizing hysteroscopic image features and clinically optimized data, we harnessed the ensemble predictive potential. Through an exhaustive grid search encompassing 140 hyperparameter combinations, we fine-tuned the XGBoost model (Appendix PP 10–11, Supplemental Digital Content 2, http://links.lww.com/JS9/C25). Hyperparameters, including the maximum depth, minimum child weight, and L1 regularization, were systematically adjusted to enhance the model performance^17,19,21^. The time-ROC curve (TimeROC package in R, version 3.6.2) was used to evaluate the accuracy of the model at different time points.

#### Application development and evaluation

We developed an independent multimodal learning application. This application seamlessly integrated our optimized AI model using Pyinstaller to ensure portability and ease of distribution. The application is available at the Github repository^22^. The performance was evaluated using the C-index and area under the curve (AUC) (R version 3.6.2) and compared with those of alternative models (Deepsurv, ElasticNet, and hysteroscopic image/EMR-based model) and clinical scores (AFS, CSGE). Decision curve analysis, which displays standardized net benefit estimates, is valuable for evaluating clinical model applications and can be used for model comparisons. Additionally, the multimodal learning application was assessed through logistic regression and stratified analysis to triage potential ART beneficiary populations and gage its patient stratification capability.

### Sample size calculation

The statistical robustness of the derived results was upheld by the attainment of statistical power exceeding 90% across three distinct datasets, all at a significance level of 0.05 (Appendix PP 12–14, Supplemental Digital Content 2, http://links.lww.com/JS9/C25).

### Role of the funder

The funders had no role in the design and conduct of the study; collection, management, analysis, and interpretation of the data; preparation, review, or approval of the manuscript; and decision to submit the manuscript for publication.

## Results

### Patient baseline characteristics

A total of 753 patients were included in the analysis, with follow-up durations ranging from 12 to 48 months. Among the patients, 322 (42.76%) achieved ongoing pregnancies following hysteroscopic adhesiolysis. Of 222 patients who underwent ART, 157 (70.7%) had ongoing pregnancies. Across the three study groups, no statistically significant differences were observed in baseline characteristics, including age, gravidity, and preoperative endometrial thickness (Table 1).

**Table 1:** Overview of the demographics and other characteristics of the recruited patients.

Characteristics	Training dataset	Validation dataset	Test dataset	*P* ^a^
Age				
≥40	41	7	24	0.115
<40	408	105	168	
Symptom duration [Months, Median (quartile)]	24 (9–48)	24 (11–36)	24 (11–36)	0.768
Menstrual pattern				
Normal	129	33	69	0.631
<1/2	107	27	37	
Hypomenorrhea	187	45	73	
Amenorrhea	26	7	13	
Age at menarche [Median (quartile)]	13 (12–14)	13 (13–14)	13.5 (13–14)	0.212
Menstrual volume before endometrial injury				
Heavy	89	28	44	0.708
Moderate	344	81	143	
Less	16	3	5	
Endometrial thickness				
≥7 mm	177	35	60	1
<7 mm	272	77	132	
Gravidity [Median (quartile)]	2 (1–3)	2 (1–3)	2 (1–3)	0.222
Parity [Median (quartile)]	0 (0–0)	0 (0–0)	0 (0–0)	0.107
Missed abortion [Median (quartile)]	0 (0–2)	0 (0–2)	1 (0–2)	0.083
Cesarean delivery [Median (quartile)]	0 (0–0)	0 (0–0)	0 (0–0)	0.039
Uterine aspiration [Median (quartile)]	0 (0–1)	0 (0–1)	0 (0–1)	0.986
Spontaneous abortion [Median (quartile)]	0 (0–0)	0 (0–0)	0 (0–0)	0.996
Uterine volume [Median (quartile)]	31.38 (25.104–41.84)	31.38 (25.104–41.84)	0 (0, 0)	0.658
BMI [kg/m², Mean (SD)]	21.78 (19.78–24.03)	21.66 (19.833–24.483)	21.93 (19.833–24.483)	0.841
AFS [Median (quartile)]	8 (5–10)	8 (5–10)	7 (5–10)	0.189
CSGE [Median (quartile)]	11 (9–15)	12 (9.75–14)	12 (9.75–14)	0.511
Ongoing pregnancy [*n* (%)]	184	58	80	0.301
ART [*n* (%)]	129	34	59	0.593
Missed visit [*n* (%)]	47	6	11	1

a Comparison between groups was performed by *χ*^2^, Wilcox, and *t*-test, respectively.

### Establishing and applying the multimodal learning model

#### Relative importance of clinical predictive indicators

**Figure 2:**
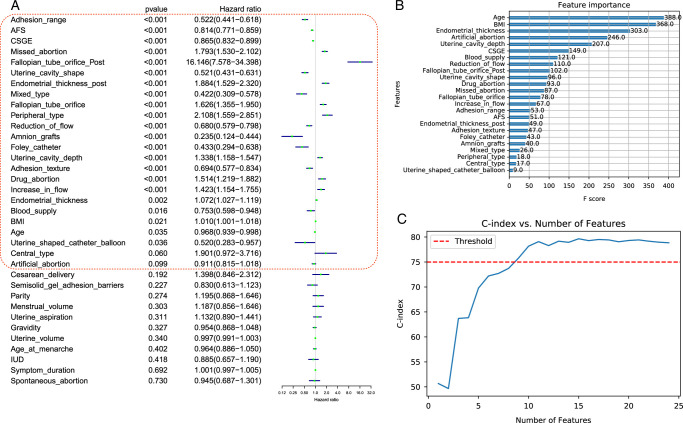
(A) The clinical predictive indicators screened based on uni-Cox regression in the training dataset, sorted by *P*-values, and the top 24 factors were selected. (B) Relative variable importance plot: 24 predictors from EMR categories ranked by relative importance to ongoing pregnancy in the training dataset. (C) C-index for prediction of ongoing pregnancy for 24 models including the top 1 to top 24 predictors of ongoing pregnancy. C-index, Concordance index.

The clinical predictive indicators were selected by conducting univariate analysis of the training dataset. Of the 35 clinical indicators, 24 had *P*-values <0.1 and were included in the analysis (Fig. 2A). The most prominent predictors of conception, ranked by the relative importance of the Xgboost algorithm, were age, BMI, endometrial thickness, history of artificial abortion (times), uterine cavity depth, CSGE score, blood supply, flow reduction, fallopian tube orifice, and uterine cavity shape (Fig. 2B). Refitting the final model with the first 1–24 predictive variables, in order, indicated a rapid increase in the C-indices as the addition of the first 10 variables (Fig. 2C). However, the including additional predictive factors did not yield significant increases in the C-index (*P*>0.05). Factors, such as AFS scores and postoperative intrauterine barriers, were also important for patient conception outcomes. However, the inclusion of the additional predictive factors did not significantly enhance the predictive performance of the model.

#### Image feature extraction

We employed the convolutional neural network of the MobilenetV3 framework to extract features from hysteroscopic images. After training, the model achieved an f1-score of 0.84 using the validation dataset. Through this process, 256 features were abstracted from the images.

#### Integration of multimodal learning

**Figure 3:**
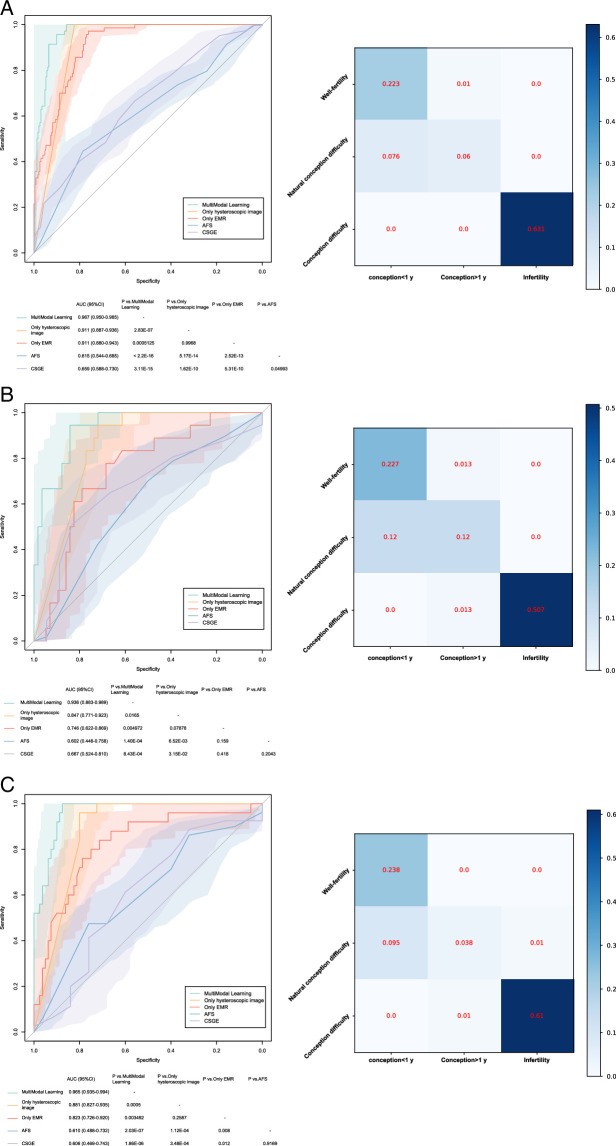
ROCs and Confusion Matrices for conception prediction in the training (A), validation (B), and test (C) datasets. The corresponding values of AUCs for multimodal learning, hysteroscopic image model, EMR model, AFS, and CSGE are shown in the table below the ROCs. AUC, area under the curve; EMR, electronic medical records.

We integrated 10 important clinical predictive features with the extracted image features. The multimodal learning model was established using XGBoost, and hyperparameter tuning was performed; the results are shown in Appendix PP 15 (Supplemental Digital Content 2, http://links.lww.com/JS9/C25). Figure 3A, B show the performance of the multimodal learning and single-modal models based on either hysteroscopic images or EMR only in the training and validation datasets. The multimodal learning model achieved higher AUCs than the single-modal models in predicting conception within 1-year (hysteroscopic images and EMR vs. multimodal learning, *P*=0.0165 and 0.00497, respectively). The multimodal model AUCs were 0.967 (95% CI; 0.950–0.985) in the training dataset and 0.936 (95% CI; 0.883–0.989) in the validation dataset, which were significantly higher than those of the AFS and CSGE scores (all *P*<0.05). The confusion matrix of the multimodal learning model showed accuracies of 0.90 and 0.85 in the training and validation datasets, respectively.

#### Development of the multimodal learning application

We developed a user-friendly AI-predictive application for multimodal learning. As shown in the Supplementary Video (Supplemental Digital Content 3, http://links.lww.com/JS9/C26), this application requires the input of 10 clinical research results and the selection of images for analysis, followed by feedback. The average analysis time per patient was 3.7±0.8 s.

### External validation of the multimodal learning application

The multimodal learning application was externally validated using an independent test dataset. The performance of the multimodal learning application in the test dataset is illustrated in Figure 3C, which shows both the ROC and confusion matrices. The multimodal learning application achieved a high AUC of 0.965 (95% CI; 0.935-0.994) for predicting conception within one year, which was significantly higher than those of traditional IUA clinical scores [AFS score AUC=0.610, 95% CI: 0.488–0.732; CSGE score, AUC=0.606, 95% CI: 0.469–0.743 (all *P*<0.05)]. It also outperformed the models based on either hysteroscopic images or EMR alone (all *P*<0.05). The confusion matrix showed that the multimodal learning application had an accuracy of 0.87 in the test dataset.

### Multimodal learning outperforms other models in predicting conception outcomes

**Figure 4:**
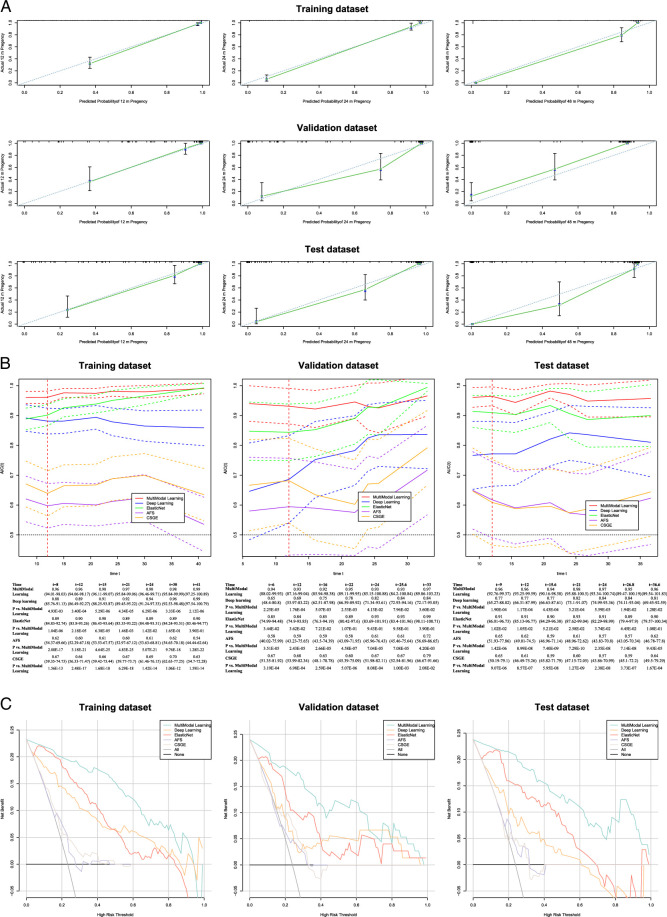
(A) Calibration plots for the multimodal learning application in the training, validation, and test datasets. (B) Time-dependent AUC curves of multimodal learning (red), Deepsurv (blue), ElasticNet (green), AFS (purple), and CSGE (orange) in the training, validation, and test datasets. (C) DCA in the training, validation, and test datasets. The *X*-axis represents the threshold probability for fertility outcome; the *Y*-axis represents the net benefit. The net benefit of all models was larger over the range of clinical thresholds compared to the reference model. AUC, area under the curve; DCA, decision curve analysis.

The calibration plot shows the predictive performance of the multimodal learning system across the three datasets and illustrates the calibration bias and deviation between the model predictions and actual events. The actual conception outcomes at 12, 24, and 48 months were highly consistent with the predictions of multimodal learning model, with most points closely aligned with the 45-degree line (Fig. 4A). The C-index, which corresponded to the calibration plot, reflected the overall predictive performance of the model. It was used to compare the predictive capabilities of the multimodal learning system with those of two other machine learning models: DeepSurv and ElasticNet. In the training dataset, the performance of the multimodal learning system significantly surpassed those of DeepSurv and ElasticNet (C-index for multimodal learning vs. DeepSurv vs. ElasticNet = 0.94; 95% CI: 0.92–0.95 vs. 0.85; 95% CI: 0.81–0.88 vs. 0.88; 95% CI: 0.85–0.91 (all *P*<0.001). In the validation and test datasets, the multimodal learning system also demonstrated significantly superior predictive abilities compared to those of the other two models (all *P*<0.01; Table 2).

**Table 2:** Comparison of concordance indexes for each assessment method.

Dataset	Indicators	C-index (95% CI)	*P* vs. Multimodal learning
Training dataset	Multimodal learning	0.94 (0.92–0.95)	
	Deep learning	0.85 (0.81–0.88)	3.46E-11
	ElasticNet	0.88 (0.85–0.91)	4.57E-09
	AFS	0.62 (0.56–0.69)	2.79E-19
	CSGE	0.66 (0.6–0.71)	3.45E-21
Validation dataset	Multimodal Learning	0.9 (0.85–0.94)	
	Deep learning	0.72 (0.63–0.8)	4.14E-07
	ElasticNet	0.84 (0.77–0.9)	0.001126
	AFS	0.61 (0.47–0.74)	1.57E-05
	CSGE	0.64 (0.53–0.74)	2.56E-06
Test dataset	Multimodal learning	0.93 (0.9–0.95)	
	Deep learning	0.76 (0.69–0.83)	1.59E-07
	ElasticNet	0.88 (0.84–0.93)	0.000726
	AFS	0.61 (0.5–0.72)	1.18E-07
	CSGE	0.6 (0.49–0.71)	2.64E-08

Time-dependent AUCs, which reflected the model’s predictive efficacy for conception at various postoperative time points, are shown in Figure 4B. Across all three datasets, the multimodal learning system achieved consistently higher AUC values than DeepSurv (multimodal learning vs. DeepSurv, *P*=3.4×10^-04^, 1.76×10^-04^, and 1.17×10^-04^ in the training, validation, and test datasets, respectively) and ElasticNet (multimodal learning vs. ElasticNet, *P*=2.18×10^-05^, 0.036, and 0.011 in the training, validation, and test datasets, respectively) for predicting conception within 1-year throughout the entire follow-up period.

The decision curve analysis for the multimodal learning and other models are shown in Figure 4C. Selecting patients for an intervention based on multimodal learning resulted in a substantial improvement in net benefit, ranging from ~0.176 to 0.208 in the training, validation, and test datasets, compared with treating all or none at the threshold probabilities. In addition, the net benefit of multimodal learning surpassed those of DeepSurv, ElasticNet, and the IUA clinical scores (AFS and CSGE).

### Stratification analysis for ART benefit ratio

**Figure 5:**
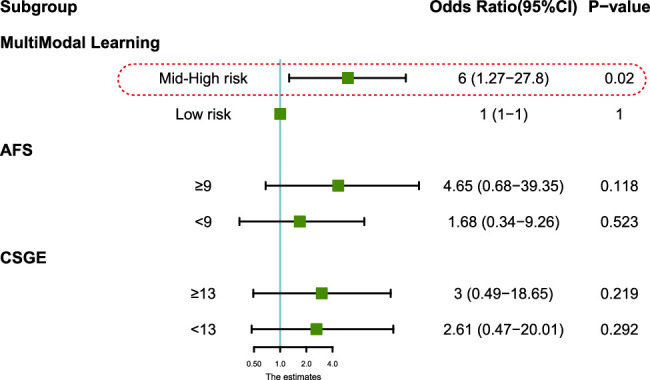
ART intervention conception odds rate from stratification analysis of the multimodal learning application and clinical score systems, including AFS and CSGE. ART, assisted reproductive technology.

To further assess the benefits of ART, patients were stratified based on the cutoff values of the multimodal learning application and AFS and CSGE scores. As depicted in Figure 5, after stratification, multimodal learning effectively distinguished the ART beneficiary group, with an odds ratio of 6 (95% CI: 1.27−27.8) in mid-high-risk patients—those with lower fertility scores, indicating reduced natural conception potential. This finding indicated a significant increase in the likelihood of conception in patients undergoing ART within this group (*P*=0.02). Conversely, significant advantages were not statistically discerned in low-risk patients demonstrating good natural conception potential or when stratified using traditional clinical scoring systems, including the AFS and CSGE scores (*P*>0.05).

### Interface and output results of the multimodal learning application

**Figure 6:**
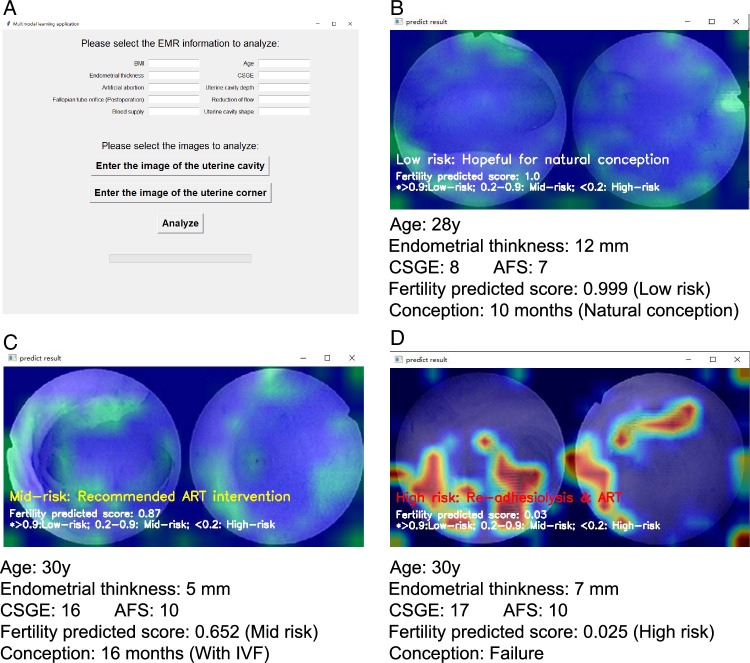
Interface of the multimodal learning application (A) and its outputs, showcasing three randomly selected cases: spontaneous conception (B), successful pregnancy post-ART (C), and pregnancy failure (D). The system classifies input data into low-risk, mid-risk, and high-risk categories, offering tailored treatment recommendations. ART, assisted reproductive technology.

The interface and output results of the multimodal learning application are shown in Figure 6. To better understand the points of interest of the model, we visualized the ROIs within the application. Three randomly selected representative samples demonstrated the focus of the model on the ROIs. The program generated visual localization maps through convolutional operations, thereby revealing areas of emphasis in the model. The model activated the regions with the most sensitive responses, highlighting features such as endometrial loss and fallopian tube blockage (shown in red), which were associated with a higher likelihood of conception difficulty. Conversely, regions such as the unobstructed fallopian tube ostia and a healthy endometrial state were emphasized in individuals more likely to conceive postoperatively (shown in green).

The fertility prediction score was derived based on the probability of conception. A score >0.9 indicated a lower subfertility risk, reducing the probability of expectant therapy failure. This guided our ART triage recommendation approach.

## Discussion

This study focused on the development of a multimodal learning model by integrating EMR and hysteroscopic images. The model was translated into a user-friendly application to predict postoperative conception outcomes in patients with IUAs, thereby assisting in their reproductive management and offering potential clinical utility. To the best of our knowledge, these aspects have not been previously reported.

Since the inception of hysteroscopic examinations, the incidence and diagnosis of IUAs has increased, particularly among women of reproductive age. In developing countries, 77.9% of patients have a history of dilation and curettage during early pregnancy, making IUAs the second most prevalent intrauterine pathology with adverse implications for female fertility^2^. Prior research has predominantly concentrated on preoperative IUA scoring and surgical interventions, while investigations into postoperative assisted reproductive conditions and optimal timing remain limited^7,11^.

According to Hooker *et al*.^23^, the IUA severity significantly reduces postoperative fertility by 24% and extends the time required to achieve conception and successful birth by a factor of 2.94. This effect is particularly pronounced in cases of moderate-to-severe IUAs. For individuals encountering challenges in natural conception, the implementation of appropriate assisted reproductive criteria has emerged as the optimal strategy for enhancing IUAs pregnancy rates while simultaneously optimizing treatment efficiency and resource allocation^8,9^. This study effectively bridges ART and predictive modeling, thereby comprehensively advancing research on IUAs.

The IUA scoring system was developed to clinically assess the severity of IUA and aid in predicting pregnancy outcomes. Currently, various assessment systems exist, including the AFS and the CSGE scores^13^. The AFS score encompasses variables such as adhesion area, type of adhesion, and menstrual pattern. The CSGE score expands upon the AFS score by incorporating indicators such as prior uterine procedures, pregnancy history, and endometrial thickness^12^. The subjectivity of these scoring systems often results in significant discrepancies in accuracy, with AUCs reaching a minimum of only 0.6, according to previous studies^2,10,14^. Furthermore, practical application models for such scoring in the post-ART management of patients are lacking. The multimodal learning model employed in this study represents a recent and prominent direction in the development of medical AI^15^. By integrating EMR and hysteroscopic images, it objectively and comprehensively evaluates patient conditions, thereby effectively enhancing the predictive accuracy.

Integrating EMR and hysteroscopic images provides a comprehensive and multifaceted representation of patient data. In our previous studies, EMR information, which encompassed crucial factors such as age, BMI, and endometrial thickness, played a pivotal role in fertility assessment^10,24,25^. The analysis in this study confirmed that the EMR information selected from the training dataset had similar predictive capabilities across the training, validation, and test datasets. This indicates the stability and suitability of the features selected to construct the multimodal learning system. Additionally, the significance of second-look hysteroscopy as a key indicator of conception outcomes has been reported^1,4^. This approach capitalizes on the strengths of both modalities, resulting in a nuanced understanding of the intricate interplay between clinical factors and visual evidence. The model provides a more profound contextual understanding through the synergistic fusion of these diverse sources, potentially capturing subtle yet impactful nuances that single-modal assessments may overlook^17^. Our study observed that in comparison to hysteroscopic images or EMR in isolation, the multimodal system demonstrates a significantly superior overall predictive efficacy.

Furthermore, by employing MobilenetV3 for image feature extraction and integrating XGBoost, a robust gradient boosting algorithm, we introduced sophisticated dimensions to our analysis. MobilenetV3 excels in extracting high-level features from images, enhancing the model’s capacity to discern intricate patterns and subtle variations^26^. This, combined with the fusion of XGBoost, capitalizes on the complementary nature of clinical and image-derived features, effectively mitigating the limitations of each standalone modality^21^. Consequently, the model acquires enhanced discriminatory power, enabling more accurate predictions of postoperative conception outcomes. This ensemble approach empowers the model to leverage the strengths of both domains, fostering a comprehensive predictive framework that surpasses the predictive capabilities of the individual components. Our study further affirms the superiority of this framework’s constructed model over the neural-network-based Deepsurv and ElasticNet models.

**Figure 7:**
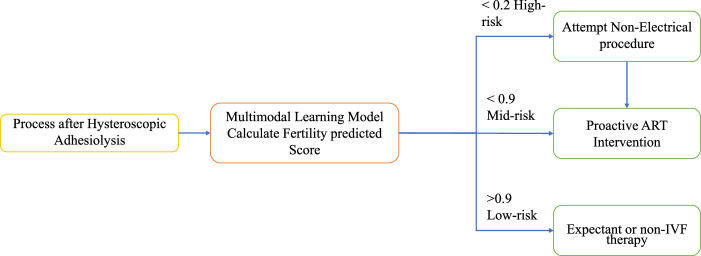
Flowchart of the multimodal learning application utilized in clinical settings.

The application schema of the system is illustrated in Figure 7. To select potential candidates for ART, this study employed a cutoff of natural conception within 1-year for population stratification. This approach stems from our clinical practice, in which patients are typically given an approximately 1-year trial period for natural conception. Additionally, based on previous research, 80% of individuals achieve natural conception within a year. Patients who exceeded this time frame were considered for ART. Identifying and screening such groups early, particularly women of advanced reproductive age, is beneficial for saving time and money. Accordingly, we recommend proactive ART intervention when the fertility score predicted by our multimodal learning model is <0.9. However, for fertility scores <0.2, which often indicate extensive readhesions, attempting nonelectrical readhesiolysis followed by observation is advised before considering ART, once the normal anatomical structure is restored.

### Limitations

Multimodal learning necessitates the analysis of diverse data types encompassing voluminous parameters; extensive sample sizes, frequently tens of thousands of samples, are often required for robust model development, which surpasses the capabilities of many medical research endeavors. This study addresses this issue using two key strategies. First, a multicenter dataset was used to mitigate potential data bias associated with single-center studies. Additionally, during the image feature training, we incorporated weights derived from the ImageNet dataset, which comprised 14 197 multiclass images. This step ensured consistent training parameters within the initial variant of the model, with a statistical power exceeding 90% across the three datasets. Future investigations will involve further augmenting the sample size to comprehensively evaluate the generalizability of the model.

## Conclusion

In conclusion, we developed a multimodal learning application system based on hysteroscopic images and EMR to predict pregnancy outcomes in patients with IUAs. The capability of the system to accurately predict natural conception in patients and provide precise postoperative risk stratification for this condition has been demonstrated. This holds potential as a valuable reference for the precise management of postoperative IUA, striking a balance between patient outcomes, time considerations, and costs, thus demonstrating promising potential for clinical application.

## Ethical approval

The study was approved and supervised by the Research Ethics Committee of the Beijing Obstetrics and Gynecology Hospital. The Chinese Ethics Committee of Registering Clinical Trials approved the revised version (protocol number; IEC-B-03v01-FJ1 and ChiECRCT20220180). Each participant had provided written informed assent for the Collection and Application of Clinical Sample and Medical Data certified.

## Consent

Written informed consent was obtained from the patient for publication of this case report and accompanying images. A copy of the written consent is available for review by the Editor-in-Chief of this journal on request.

## Sources of funding

This study was financially supported by the National Key Research and Development Program of China (2018YFC1004803).

## Author contribution

B.L., H.C., and H.D.: designed the study and developed the conceptual ideas; B.L. and X.L.: collected all the input sources and additional data and annotated the images; B.L. and H.C.: implemented the main algorithms and other computational analysis and analyzed the results; B.L., X.L., and H.D.: have accessed and verified all the data in the study; H.D.: had final responsibility for the decision to submit for publication.

## Conflicts of interest disclosure

Not applicable.

## Research registration unique identifying number (UIN)

ClinicalTrials.gov; NCT05381376.

## Guarantor

Hua Duan.

## Data availability statement

The application code is publicly shared on github, https://github.com/libohan1110/Multimodal-Learning.git, for peer review and academic exchange. The raw data supporting the conclusions of this article will be made available on request from the corresponding author, without undue reservation.

## Provenance and peer review

Not applicable.

## Supplementary Material
